# Fabrication of Parylene-Coated Microneedle Array Electrode for Wearable ECG Device

**DOI:** 10.3390/s20185183

**Published:** 2020-09-11

**Authors:** Afraiz Tariq Satti, Jinsoo Park, Jangwoong Park, Hansang Kim, Sungbo Cho

**Affiliations:** 1 Department of Electronics Engineering, Gachon University, Seongnam-si, Gyeonggi-do 13210, Korea; afraiztariq91@gc.gachon.ac.kr; 2 Gachon Advanced Institute for Health Science and Technology, Gachon University, Incheon 21999, Korea; jspark88@gc.gachon.ac.kr; 3 Department of Mechanical Engineering, Gachon University, Seongnam-si, Gyeonggi-do 13210, Korea; jwbark21@keti.re.kr (J.P.); hskim70@gachon.ac.kr (H.K.)

**Keywords:** microneedle electrode, ECG, cardiovascular diseases, parylene, impedance

## Abstract

Microneedle array electrodes (MNE) showed immense potential for the sensitive monitoring of the bioelectric signals by penetrating the stratum corneum with high electrical impedance. In this paper, we introduce a rigid parylene coated microneedle electrode array and portable electrocardiography (ECG) circuit for monitoring of ECG reducing the motion artifacts. The developed MNE showed stability and durability for dynamic and long-term ECG monitoring in comparison to the typical silver-silver chloride (Ag/AgCl) wet electrodes. The microneedles showed no mechanical failure under the compression force up-to 16 N, but successful penetration of skin tissue with a low insertion force of 5 N. The electrical characteristics of the fabricated MNE were characterized by impedance spectroscopy with equivalent circuit model. The designed wearable wireless ECG monitoring device with MNE proved feasibility of the ECG recording which reduces the noise of movement artifacts during dynamic behaviors.

## 1. Introduction

Cardiovascular disease (CVD) is the leading cause of death globally. More than 17.6 million people lost their life in 2016, and the number is expected to grow by over 23.6 million by the year 2030. The healthcare system also suffers a major economic burden directly (e.g., hospitalization, physician visits, rehabilitation services, and drugs) and indirectly (e.g., long or short-term disabilities and premature mortality can cause losses in productivity) [1]. Furthermore, the risk factor for mortality and morbidity due to the novel infectious coronavirus disease (COVID-19) is higher for people with chronic health conditions. The pandemic COVID-19 has also increased the demand for remote health-monitoring systems, especially for CVDs [2,3]. Sudden cardiac death and other heart problems can be avoided by using suitable prevention techniques, such as long-term monitoring of the heart signal and taking appropriate medication to prevent critical conditions [1]. Therefore, an efficient long-term electrocardiography (ECG) monitoring technique is required to improve the accuracy of diagnosis of arrhythmia.

In recent years, significant work has been done to develop skin-mountable electronic devices and suitable electrodes to monitor the physiological status. Traditional wet Ag/AgCl electrodes have been mostly used for bioelectric signal recording because they have low electrode interfacial impedance and are good for monitoring high-resolution bioelectric signals with low noise [4]. However, several drawbacks limit their additional applications, such as the following: (1) The Ag/AgCl electrodes require special skin treatment, such as skin abrasion, hair cutting, and electrolytic gel coating. (2) Previous studies reported that the electrolytic gel used for wet electrodes may cause allergic reactions and skin irritation in some patients [5,6,7]. (3) The electrolytic gel gradually dries after some time and causes noise in the collected signal, which hinders its application for long-term bio-signal monitoring [5,7]. Dry electrodes are an alternative for long-term bio-signal monitoring because they do not use electrolytic gel and do not exhibit degradation in the signal quality and are convenient to use while requiring less preparation time [8]. However, the measurement of bioelectric signals using dry electrode is vulnerable to variable skin interfacial impedance and motion artifacts resulting from different contact conditions between the electrode and skin [9,10].

**Figure 1:**
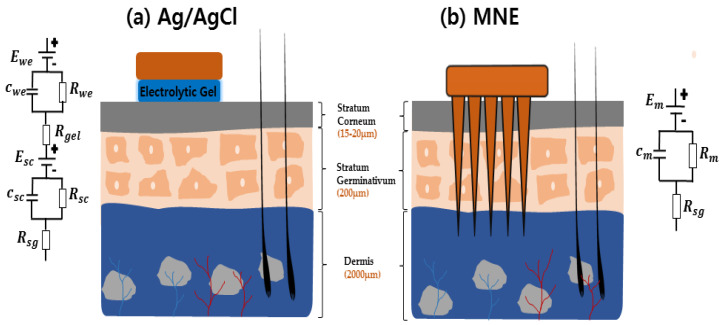
Schematic of the interface between (**a**) the skin and typical Ag/AgCl electrode, (**b**) the skin and microneedle array electrode (MNE).

Recent studies have indicated that MNEs can be a good alternative to address the above mentioned problems in dynamic and long-term bio-potential monitoring. First, MNE provide low electrode-skin contact impedance by puncturing through the skin and eliminating the outermost skin layer, i.e., the stratum corneum (SC), and makes direct contact with the epidermal layer or some part of the superficial dermis layer as shown in Figure 1b [5,11,12]. Gill et al. studied about the pain sensation from needle height and discussed that the participant reported no pain or minimal pain by the needle length under 700 μm [13]. Further, the nerves and blood vessels are located at the end of dermis layer. The microneedles do not reach the nerve index because of their small size. Thus, the puncture process is painless [14,15]. Second, small motion artifacts are achieved because of the stable contact interface between the MNE and skin. Third, the risk factors for skin infection or skin irritation in some patients resulting from the Ag/AgCl gel can be eliminated because no skin treatment is needed for MNE [16]. Further, MNE provides a good result for dynamic and long-term bio-potential monitoring compared with the Ag/AgCl electrode. During movement, an arm swing causes a skin shift in the patient, which results in movement artifacts in signal due to the relative position drift between the Ag/AgCl electrode and skin [17,18,19]. Additionally, in long-term bio-signal monitoring, the gel used in Ag/AgCl electrode dries after some time, introducing noise in the signal [19]. Both these problems can be avoided using MNE because it maintains a stable skin-electrode interface by grasping the skin with microneedles and no gel is needed [5,7,19]. These characteristics have attracted the attention of researchers to further investigate MNEs for bioelectric signal monitoring.

The issues of skin infection and microneedle breakage inside skin during MNE penetration were considered. Microorganisms can enter the skin through the open channels made by MNE. A study from Donnelly et al. stated that the puncture resulted from the microneedles are lower than the hypodermic needle puncture, and no microbial penetration to the epidermal skin in case of microneedles puncture. Considering skin antimicrobial properties, the appropriate application of microneedle to the skin should not cause skin infection or irritation in normal conditions [20,21]. The skin healing process of the pores created by the microneedle is in the order of hours on timescale. The healing time in case of microneedle pore is lower than the healing time required for the hypodermic syringe pore, which further reduces the infection risk [5].

Based on the results, the breakage of the needle was not observed under the maximum force of 35 N, which is enough to cause the serious tissue damages. Although the accidental breakage of the needle happens, the medical grade 316L stainless steel is not toxic or inflammatory and has been used in clinical practices [22,23]. Additionally, Given the elastic nature of the skin, there is an upward component of force acting on the slope wall of the needle that assists in pushing out the broken needle from the skin. Moreover, the outer dead skin cell renewed after 2–3 weeks which helps in removal of outside particles [5]. Despite all, the breakage of microneedle inside skin and its potential risk due to foreign body granulomatous response of skin need further investigation.

The electrical models of both MNE and wet Ag/AgCl electrode are shown in Figure 1a,b, respectively. The wet-electrode model includes a battery *E*_we_ represents half-cell potential. Capacitance *C*_we_ and resistance *R*_we_ represent the interface between the Ag/AgCl electrode dressing and gel. Resistor *R*_gel_ indicates the electrical resistivity of the used gel. SC is similar to a semipermeable membrane. The difference between the ion concentration of the electrolytic gel and SC creates a potential difference, which is denoted as *E*_sc_. The SC layer shows capacitive and resistive behavior trends, which are denoted as *C*_sc_ and *R*_sc_, respectively. Finally, the stratum germinativum (SG) layer and tissues also show resistance *R*_sg_. Comparatively, the MNE model shown in Figure 1b is simpler. *E*_m_ indicates the half-cell potential of the MNE. Because the conductive needles directly contact the SG layer, they create (*C*_m_ and *R*_m_) an electrode–electrolyte interface. The SG layer resistivity is denoted as *R*_sg_ [12].

Various approaches for MNE fabrication and recording performance were reported in the last decade. However, a recent review has suggested that more work needs to be done to improve the MNE fabrication process, mechanical strength, and signal quality [24]. Furthermore, various geometric dimensions, such as electrode wall thickness, number of arrays, needle height, and interspacing, could be adopted to increase the performance graph of MNE [16,24]. The previously fabricated MNEs were round-shaped, and wall thickness used is approximately 0.3 mm [5,6,25]. Rigid round-shaped electrodes do not conform to a moving curvature skin [17,26]. Chen K et al. [6]. showed dynamic ECG signal recorded with a round shaped rigid electrode. The reported dynamic ECG signal shape need further improvement. Different electrode wall thicknesses and shape can be used to increase the fidelity of MNE for dynamic ECG recording. Second, to the best of our knowledge, earlier studies did not discuss the impedance that occurs due to the direct contact between MNE wall and skin. Additionally, Lei Ren et al. [16] stated that smaller number of microneedles can be easily inserted into the skin because it requires a low insertion force. In addition, Gill et al. reported that pain intensity can be minimized by using small number of microneedles [13]. Therefore, it was needed to fabricate a microneedle which can address the above problems. First, fabricating an MNE with the possible least number of microneedles to minimize the pain sensation. Second, the MNE can be coated with parylene to reduce its contact with the outermost layer of the skin. Furthermore, a minimal wall thickness and a rectangular shape can allow MNE to be flexible and adjust to the skin movement which results in good signal during dynamic ECG monitoring.

In the present study, a novel square-shaped parylene-coated MNE array (3 × 5) electrode is introduced that is fabricated using a simple fabrication technique. The MNE is insulated with 5 μm parylene to minimize the skin-electrode contact impedance. The proposed MNE is rigid yet slightly flexible owing to its shape and small wall thickness that provide robust contact with the skin and adjust to the motion artifacts. This study discusses the MNE fabrication process, and different types of tests are performed to investigate its performance. Furthermore, a small wireless ECG monitoring system is introduced to analyze the MNE performance with minimum external interferences. The experimental results showed that the presented MNE and developed system has good potential for dynamic and long term bio-signal monitoring.

## 2. Materials and Methods

### 2.1. Materials

Medical grade 316L stainless steel was selected for its good tensile and yield strength. 316L stainless steel has been commonly used in medical field for surgical practices. The stainless steel electrode was electroplated with Gold (Au). Gold is a good biocompatible element which do not create any toxic reaction when used with internal organs or skin [27]. 316L stainless steel and gold were obtain from (Samsung metal, Incheon, Korea). Polydimethylsiloxane (PDMS) was selected because of its viscoelastic properties. Silicone elastomer curing agent and Silicone elastomer base by (dowhitech, Seoul, Korea) were used for preparation of PDMS. Commercially available Parafilm^®^ M by (Bemis^TM^, USA) was also used to cover the MNE connection pad. parylene dimer Dichloro-p-xylene by (Daisan Kasei Co., Ltd., Ichihara, Japan) was used for insulation of MNE surface area and it was selected for its good insulation and safe biological properties.

### 2.2. MNE Fabrication

**Figure 2:**
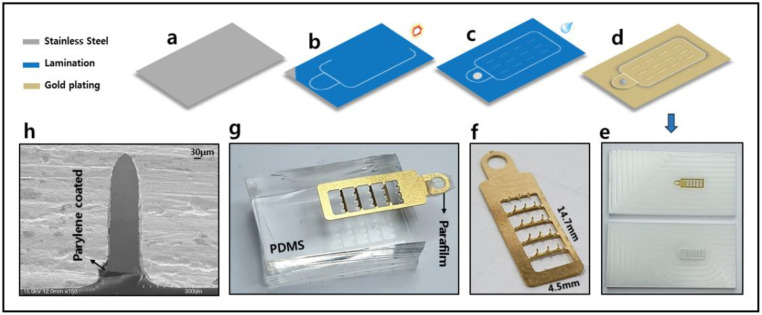
Fabrication process of MNE; (**a**) medical-grade 316L stainless steel; (**b**) lamination of light sensitive photo resist and exposure to UV light; (**c**) developing for the positive image print; (**d**) gold electroplated; (**e**) jig to move microneedles at 90 degrees; (**f**) MNE with standing microneedles; (**g**) microneedles inserted into polydimethylsiloxane (PDMS) and connecting pad covered with Parafilm M^®^ for parylene coating the surface area of MNE; (**h**) SEM image of single needle showing the parylene coating.

Figure 2 shows the overall fabrication process of the MNE. A 100 μm 316L stainless steel substrate was used for the MNE fabrication, as shown in Figure 2a. After selecting the Stainless steel substrate, a light sensitive resist was laminated on the selected substrate and was exposed to the UV light shown in Figure 2b, followed by developing to get the positive image of product shown in Figure 2c. The MNE was obtained by exposing the pattern substrate to a jet of chemical etchant, such as ferric chloride, and applied a pressure of 2 kgf/cm^2^ for 1 min. A thin film of Au layer was electroplated on the MNE surface, as shown in Figure 2d. The Au layer added biocompatibility and increased the MNE conductance [28]. Microneedles were then punched using a jig to bend them at 90°, shown in Figure 2e,f. To passivate the MNE base plate with parylene film, the microneedles were inserted into polydimethylsiloxane (PDMS), and the contact pad was covered with parafilm M^®^ as shown in Figure 2g, so only MNE baseplate got coated with the parylene film. The parylene film coating is done by chemical vapor deposition technique using parylene deposition system Lavida 110 by (Femto Science Inc., Hwaseong-si, Korea). At first, the raw solid parylene dimer is vaporized into gas by heating under vacuum. Next, the gas is pyrolized to get the monomeric form of dimer by cleaving it. In the deposition chamber, the monomer gas gets deposited on MNE exposed part as a thin polymer film. Parylene coated single microneedle is shown in Figure 2h.

The thickness and uniformity of parylene coating on MNEs is controlled by following the protocol given by the company (Femto Science Inc, Hwaseong-si, Korea). According to the protocol, the parylene film of 5 μm thickness was coated by following the procedure: weigh about 5 g of Dichloro-p-xylene, the paraylene deposition system door heater temperature was maintained at 150 °C, furnace temperature was set to 690 °C, chuck’s rotational speed was set to 3 rpm. For the uniformity of the coating, the temperature for vaporization is increased gradually.

### 2.3. MNE Parylene-Coating Test

When the MNE is inserted into the skin, the electrode wall makes contact with the outermost skin, which adds dead-skin impedance to the recorded value. Therefore, it is required to insulate the MNE surface area with parylene to minimize contact between the skin and MNE. A parylene-coating test was conducted to check the impedance of the MNE before and after the parylene coating. We used 0.9% NaCl for the test because it has the same pH and conductivity levels as the human extracellular fluids [29,30]. The MNEs were immersed in a 0.9% NaCl solution, and the other side was connected to the impedance-measurement system CompactStat potentiostat from IVIUM Technologies (Eindhoven, The Netherlands). The frequency range was set from 0 Hz to 100 kHz to investigate the impedance response of the MNE with and without parylene coating.

### 2.4. Mechanical Test of the MNE

#### 2.4.1. Fracture Test

**Figure 3:**
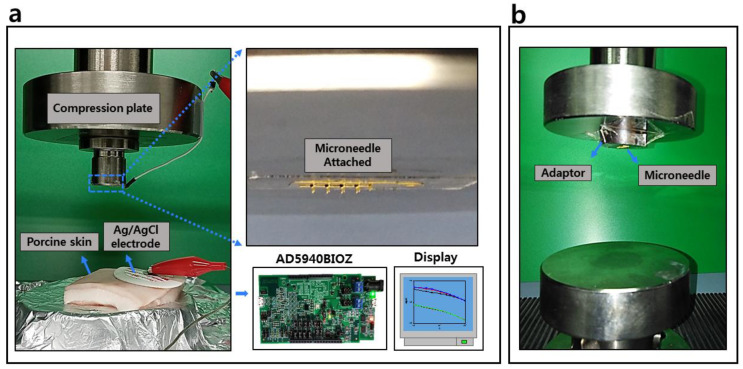
(**a**) Electrode–skin interface impedance (EII) recording setup during the insertion test. (**b**) Fracture test setup.

The mechanical strength of microneedles is one of the important factors to be addressed. Microneedles are subjected to different stresses during the insertion and removal process; thus, microneedles fracture inside the skin may occur during these processes. Hence, a fracture test was conducted to investigate the strength of microneedles under pure compression force. The test was performed using an AG-X Plus 50 Kn instrument (Shimadzu, Germany). The following process was followed in the test: (1) The compression force was guided using an adapter attached to the upper compression plate. (2) The MNE was fixed on the adapter. (3) The MNE holding compression plate was moved downward at 0.01 mm/s. (4) The displacement and loading force were simultaneously recorded. The test was conducted in a normal laboratory environment. The schematic of the fracture test setup is shown in Figure 3b.

#### 2.4.2. Skin-Puncture Test

The fabricated electrode’s microneedles were sharp and sufficiently thin to puncture the skin. A test was conducted to ensure that the microneedles penetrated the skin. First, the MNEs were placed on the forearm of 25-year-old male healthy volunteer. The MNEs were not pushed inside the skin, and the impedance was measured. The second recording was taken after pushing the MNEs inside the skin. Both MNEs were connected with AD5940BIOZ impedance-measurement system (Analog Devices, Norwood, MA, USA). A frequency ranges of 100 Hz to 10 kHz was selected. The test was conducted under normal room conditions. Same test was repeated for two other volunteers of age between 25–29. MNEs were sterilized with 70% ethanol before their application to human skin and all ethics were considered. Written informed consent was obtained from the volunteers and the study was ethically approved by Institutional review board of Gachon University (IRB No. 1044396-202008-HR-158-01).

### 2.5. Bio-Signal Monitoring

The fabricated MNE performance was further investigated by electrode–skin interface impedance (EII) and ECG tests using both MNE and commercially available Ag/AgCl electrodes. The same test conditions and electrode positioning were used for both electrodes. Three healthy volunteers of age between 25–29 were selected for the bio-signal recording tests.

#### 2.5.1. EII Measurement Test

Figure 3 shows the setup for the EII measurement test. The two-electrode method was used to record the impedance. The Ag/AgCl electrode and MNE placements are shown in Figure 3. The distance between the wet Ag/AgCl electrode and MNE was approximately 1 cm. Both electrodes were connected to AD5940BIOZ impedance-measurement system (Analog Devices, Norwood, MA, USA). The frequency range was set from 100 Hz to 10 kHz. Loading machine AG-X Plus was used to load the MNE into a porcine cadaver skin. The pork skin was placed on the lower compression plate, whereas the MNE was attached to the upper compression plate. The upper compression plate was moved downward at a speed of 0.5 mm/s, and in parallel the EII was recorded.

#### 2.5.2. MNE Animal Model Test

An animal model test was also conducted to investigate the MNE performance. All the animals were handled by the Gachon university Animal Care Center and the protocol given by the Animal care committee (animal experiment approval number: GIACUC-R2019028, Gachon University, Animal Experiment Committee) was followed during the animal model test. 

Two MNEs were inserted on the chest of a male Sprague-dawley rat model after removing the hair from the skin with surgical blade. ECG recordings were conducted to investigate the MNE performance with an animal model. Two-lead method was used to record the ECG signal. The electrodes were connected to a bio-signal measurement system (RHS2116) by (Intan Technology, Los Angeles, CA, USA). The ECG of the rat was recorded while it was in a resting position. The test was conducted under normal room conditions.

#### 2.5.3. ECG Tests (Static, Dynamic and Long-Term Tests)

ECG monitors the heart activity by placing electrodes on the skin. Static and dynamic ECG recording techniques were used to test the sensing performance of the MNE. The II-Lead method was applied to record the ECG test results. The results of the MNE and Ag/AgCl electrodes under static and dynamic conditions were recorded and compared. Static ECG signal is good for detecting arrhythmia. While, dynamic ECG signal monitoring can be used to detect other heart diseases, such as myocardial ischemia and premature beats [31]. The MNEs and wet Ag/AgCl electrodes were tested on three healthy volunteers of age between 25–29. Both electrodes were placed on the same position at the chest for all volunteers, and the same conditions were set for both tests. The electrodes were connected to a developed bio-signal system. During the static-state test, the subject sat on a chair and was in a resting position. For the dynamic test, the subject walked with a uniform speed of 4 km/h. The wet Ag/AgCl and MNEs were also tested for long-term bio-signal monitoring. Because the wet Ag/AgCl electrode used an electrolytic gel for good contact with the skin, the gel dried after some time and degraded the signal quality. For the long term ECG monitoring term, electrodes were placed on the front arm because of its easy availability. Both electrodes were tested for one week and the results were compared.

### 2.6. Two Lead Wireless ECG Measurement System Design

**Figure 4:**
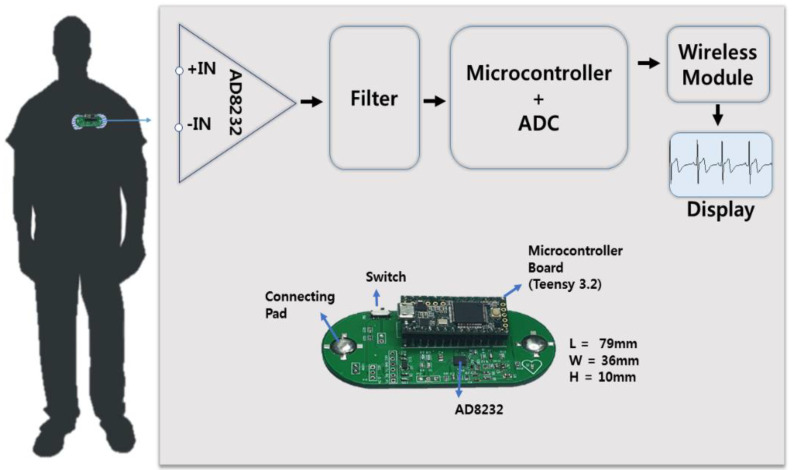
Developed wireless electrocardiography (ECG) monitoring system.

A two-lead wireless ECG measurement system was developed to investigate the performance of the MNE without any external interference due to the movement of wires. Figure 4 shows the developed ECG measurement system’s detail. An operational amplifier, i.e., AD8232 (Analog Devices, Inc., Norwood, MA, USA), based on a small integrated circuit, is used to extract, amplify, and filter the small biopotential signals. Amplifier AD8232 simultaneously applied high-pass filtering and gain. This integrated chip can reject a large electrode offset of up to ± 300 mV, and it can amplify a small signal by 100 times. AD8232 contains an RLD amplifier that improves the system’s common mode rejection. In case of two-electrode configuration, the common mode rejection can be improved by driving the input bias current through 10 MΩ resistor using the RLD amplifier. The operational amplifier-based system removed the additional noise by creating a three-pole low-pass filter. The filter cutoff frequency can be calculated using the following Equation:

*f_c_* = 100/(2π *R C*) (1)

Few passive components can be changed to adjust the gain and cutoff frequencies. In this system, the cutoff frequencies are set to 0.5 and 40 Hz. A high-resolution analog-to-digital converter (ADC) was required to acquire the output signal. Embedded microcontroller Teensy 3.2 is used because it has a high-resolution ADC, high processing speed, and low power consumption. A small Bluetooth transceiver, namely, HC-06, is used to wirelessly transmit the collected signal to the computer, and a serial plotter is used to view the ECG signal. The developed system is capable of processing and visualizing the ECG data in real time on mobile or desktop devices. 

## 3. Results and Discussions

### 3.1. Characterization of MNE

**Figure 5:**
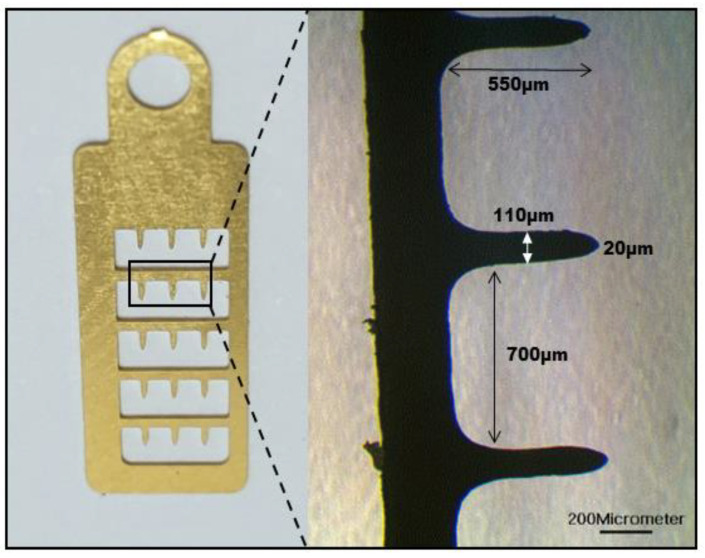
MNE dimensions taken by microscope at 200-micrometer scale.

Stainless steel was used for the MNE fabrication, and the needles were patterned on it. The coated gold layer added biocompatibility and conductance to the MNE. The MNE consisted of 3 × 5 microneedles at an interval of 700 μm. The length of the MNE is 14.7 mm, and the width is 4.5 mm. The needle height is approximately 550 μm, the diameter at the middle is 110 μm, and the radius of the tip is 20 μm. The microneedles have rectangular shape and are smooth to easily penetrate the skin. The thickness of the stainless steel is kept small (0.1 mm) to make the MNE slightly flexible so that it can adjust to the skin movement during dynamic ECG monitoring. Figure 5 shows the MNE dimensions. The microscopic image is taken by TCM 400 by (Labomed, Fremont, SF, USA).

### 3.2. MNE Parylene-Coating Test

**Figure 6:**
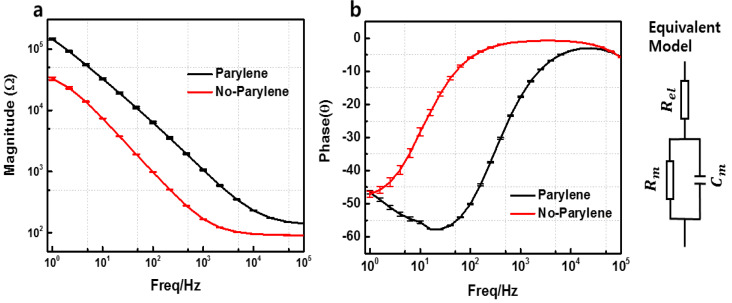
Parylene-coating test with 0.9% NaCl. (**a**) Magnitude. (**b**) Phase. The data represents the average of three independent measurements with error shown in SD (*n* = 3).

Figure 6a,b shows the comparison of impedance magnitude resulting from the MNE with and without parylene coating. Three tests were performed while average and standard deviations (*n* = 3) were calculated and plotted. The equivalent-circuit model of the electrode-electrolyte interface is also shown [24]. The impedance value of the parylene-coated electrode shown with black line is higher than the value of non-parylene-coated electrode presented by red line. In the case of the non-parylene-coated electrodes, more area was exposed to the solution. Thus, a higher number of ions were transferred to the solution, which resulted in lower impedance than the parylene-coated electrode, which had a smaller area exposed to the solution. Hence, the MNE could be coated with parylene to eliminate the contact impedance between the skin and electrode wall.

### 3.3. Fracture Test Performance

**Figure 7:**
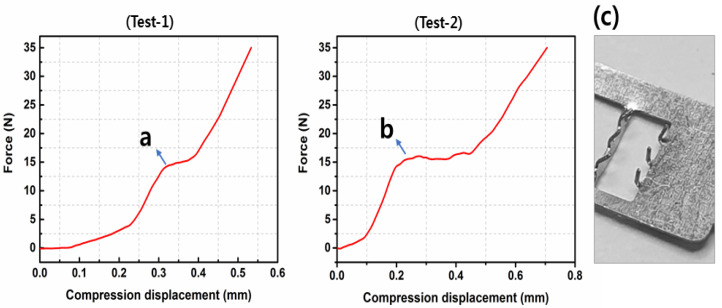
Fracture test of the MNE. (**a**,**b**) Resistance force before mechanical failure of the microneedles. (**c**) Microneedles after the fracture test.

Figure 7a,b shows the resistance force of MNE against the compression displacement. The force linearly increased with the loading displacement as the MNE tips touched the lower compression plate. The microneedles begin to deform as the loading displacement proceeds. The mechanical failure of microneedle shafts started at point “a, b”, when the compression force was almost 16 N. Hence, the MNE can be pressed up to 16 N without being reached to its mechanical failure point. It is comparable with the resistance force tested by Chen, K et al. [6]. Assume, all the microneedles showed same response during the fracture test, so resistance force for one microneedle is 1.06 N. The compression force was applied up to 35 N to check the microneedle fracture, but no fracture was observed, as seen in the Figure 7c. Thus, microneedles have good strength and toughness to prevent their breakage inside skin.

### 3.4. Bio-Signal Recording Performance

#### 3.4.1. EII Performance

**Figure 8:**
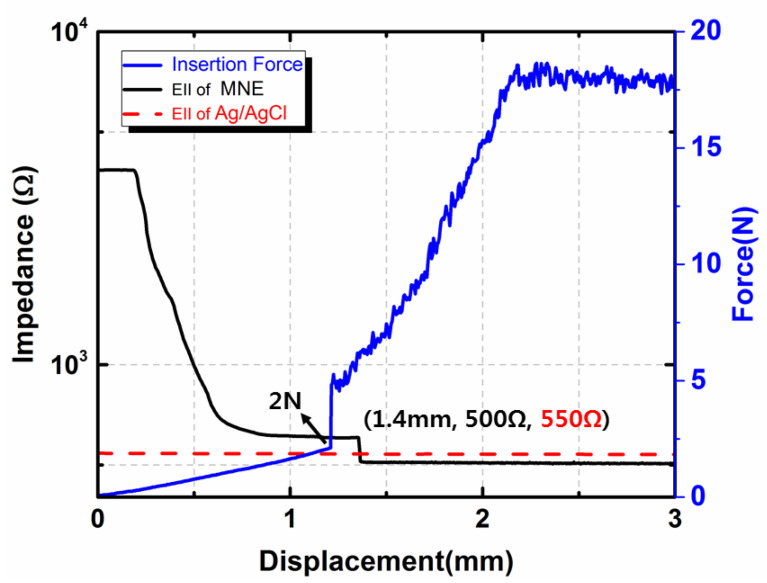
Insertion force and EII recorded during MNE insertion into the porcine cadaver skin.

Figure 8 shows the impedance and force versus displacement graph during the MNE insertion process inside the porcine cadaver skin at 500 Hz input frequency. When the test started and microneedles make contact with the skin, the insertion force was 0.08 N and the impedance was 3807 Ω. The low insertion force and high impedance values were because of the poor contact between microneedles and skin. The insertion force increased gradually and the impedance value started decreasing as the MNE tips made good contact with the skin. Initially, the force versus displacement graph was linear because the applied force of up to 2 N deformed the skin. The porcine cadaver skin resisted the insertion of the needle because of the potential energy stored in the skin. A sudden increase in the insertion force occurred after 2 N. At this point, the skin-deformation process stopped and the applied 3 N force from 2 N to 5 N was used to puncture the porcine cadaver skin. Assume that all microneedles penetrated the skin, the insertion force for one microneedle is 0.2 N. The insertion force of MNE is about an order lower than the mechanical failure resistance force of MNE. A sudden decrease in the impedance value also occurred after the 5 N insertion force, further verifying that the MNE punctured the SC layer, which eliminated the dead-skin impedance. At this point, the impedance value recorded with the MNE was lower than that recorded with the wet Ag/AgCl electrode. The selected pork skin was punctured by a 5 N force in which 2 N was used to deform the skin. This result attributed to the fact that the MNE can eliminate the dead-skin impedance by piercing through the SC layer of the skin and make direct contact with the conductive epidermis layer. Additionally, the microneedle insertion force required is lower than the microneedle yielding force shown in fracture test section, which is essential for MNE optimal design.

#### 3.4.2. MNE Performance on Animal Model

**Figure 9:**
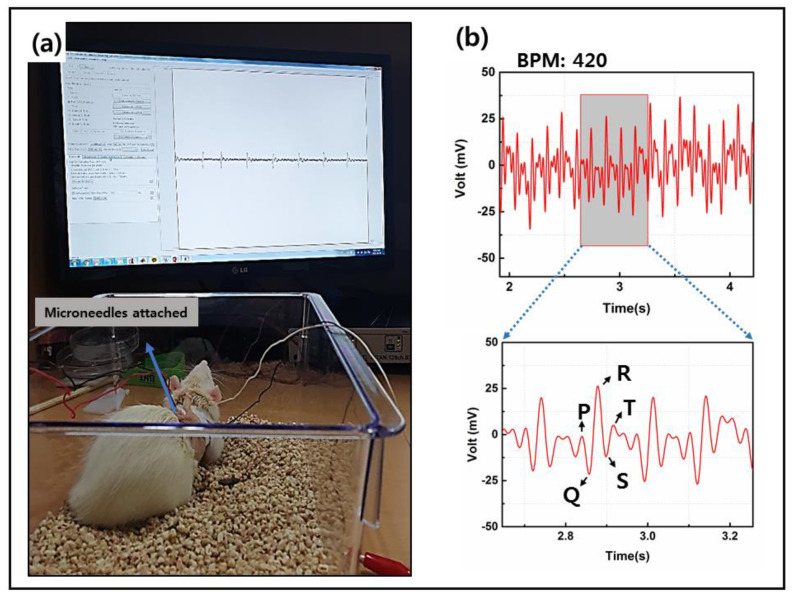
(**a**) Rat with electrodes fixed on the chest. (**b**) ECG of the rat.

Figure 9a shows the rat with the MNE inserted inside the skin. Figure 9b shows the recorded ECG signal. During the animal model test the rat was moving which causes some noise in the collected signal yet the ECG waveform can be seen in Figure 9b. The P-waves, QRS complex and T waves are distinguishable. The recorded pulse rate of the rat was 420 BPM. Fabricated MNE showed good fidelity for measuring heart signal of animal model.

#### 3.4.3. Human Skin-Puncture Performance

**Figure 10:**
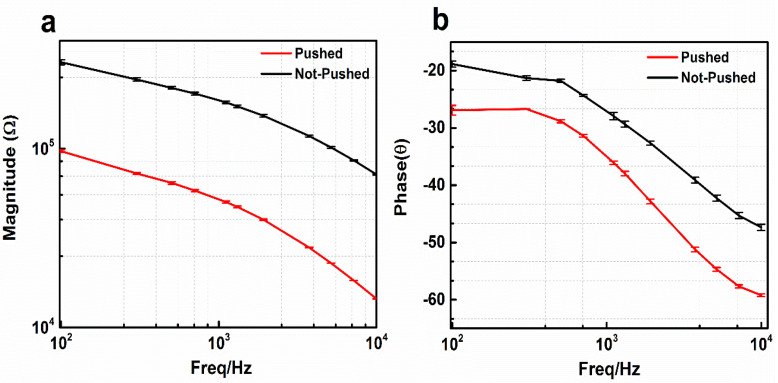
Electrical impedance spectra. (**a**) Impedance magnitude and (**b**) phase value before and after penetration of MNE inside skin. The data represents the average of three independent measurements with error shown in SD (*n* = 3).

Figure 10a,b shows the comparison of the impedance results before and after puncturing the skin using MNE. Average and standard deviations from three tests (*n* = 3) were calculated and plotted. Initially, the MNE was placed on the arm of the volunteer without any pressure. The resulting impedance value was high as shown by black line because the dead skin also contributed to the impedance value. Subsequently, some forces were applied on the microneedles to insert them into the skin. The redline show low impedance as the MNE penetrated the skin and eliminated the SC impedance. Hence, it is proven that the microneedles punctured the skin and eliminated the dead-skin impedance. Gill et al. studied the pain sensation by the MNE height and reported that 30% of the subjects feel no pain by under 700 μm microneedle length and others just reported minimal pain in comparison to the hypodermic needle [13]. During the skin puncture test, three volunteers were asked about the pain due to MNE insertion. No pain or minimal pain was reported due to the push on the MNE and the intensity of pain was lower than the intensity of hypodermic needle pain.

#### 3.4.4. ECG Static and Dynamic Tests on Human

**Figure 11:**
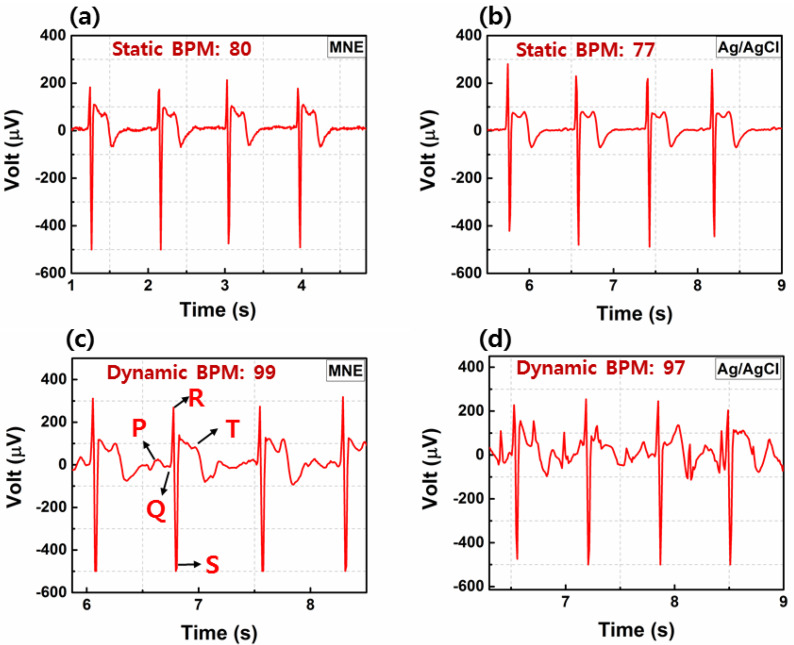
ECG using MNE compared with Ag/AgCl electrode for (**a**,**b**) static or (**c**,**d**) dynamic behaviors.

A comparison of the static and dynamic ECG signal recordings of the wet Ag/AgCl electrode and MNE are shown in Figure 11. The static ECG signal is almost similar in both electrodes, and the features, such as the P wave, QRS complex, and T wave, are all distinguishable. A small number (3 × 5) of microneedles array exhibited good fidelity in the static ECG signal monitoring. Figure 11c,d show the dynamic ECG signals recorded with both electrodes while the volunteer was walking. The possible position drift between the skin and Ag/AgCl electrode during the movement introduced noise, as shown in Figure 11d. In the traditional Ag/AgCl electrode case, only the R peaks were visible, and the baseline drift was high. Meanwhile, the potential signal recorded using MNE was clearer, and the P, Q, R, S, and T waves were more visible. Because the microneedles grasped the skin and the small (0.1 mm) wall thickness and rectangular shape allowed the MNE to adjust with the moving skin, a good-quality dynamic ECG signal was maintained, as shown in Figure 11c. we measured the ECG of three volunteers during movement and found that the noise caused by motion artifacts in ECG using MNE was lower than Ag/AgCl electrode. Therefore, the fabricated MNE is a good choice for dynamic ECG monitoring. The ECG signals were recorded for more than one minute and the BPM measured for three volunteers was in between 75–105. 

#### 3.4.5. Long-Term ECG Monitoring Comparison of the MNE and Ag/AgCl Electrode

**Figure 12:**
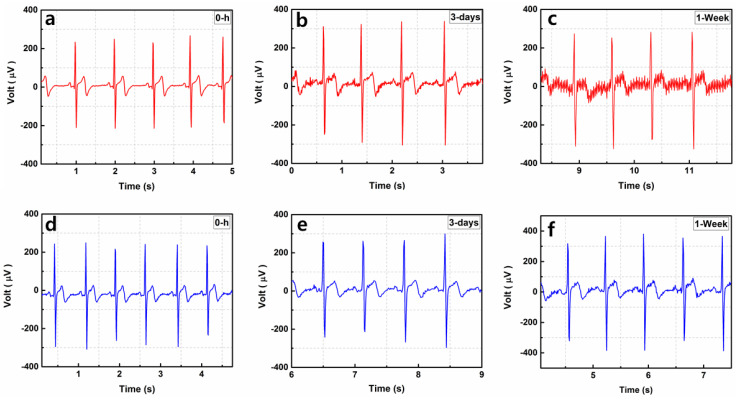
Long-term use of electrode for ECG recording with (**a**) Ag/AgCl at 0 h, (**b**) Ag/AgCl after 3 days, (**c**) Ag/AgCl after 1 week, (**d**) MNE at 0 h, (**e**) MNE after 3 days, (**f**) MNE after 1 week.

Figure 12 shows the long-term ECG signal monitoring results of the wet Ag/AgCl electrode and MNE. Figure 12a–c show the Ag/AgCl recorded signal at 0 h, after 3 days, and after 1 week, respectively. The quality of the ECG signal decreased and the baseline noise increased with time. Because the electrolytic gel used for the wet electrode dried after some time, noise was introduced to the recorded signal. The signals recorded using the MNEs were almost similar at 0 h, after 3 days, and after 1 week. This result proves that the proposed MNE is a better choice for long-term bio-signal monitoring. MNE skin retention is an important aspect for long term ECG monitoring. The skin insertion and retention performance of proposed MNE could be further improved. The emerging additive manufacturing 3D printing techniques demonstrated in recent papers could be beneficial for 3D and sharper microneedle geometries. The 3D printed arrow head microneedles can improve skin penetration and retention performance of MNE [32,33].

## 4. Conclusions

In this study, a novel square-shaped parylene-coated MNE was introduced, which showed good results for dynamic and long-term ECG signal recording. MNE was patterned on a stainless steel substrate, and a thin Au film was electroplated to ensure biocompatibility and conductance. The fabricated MNE consisted of the least number of microneedle arrays (3 × 5), which required a small amount of insertion force for skin penetration. The MNE surface was coated with parylene to minimize contact between the bare gold surface and skin. When the microneedles were inserted into the skin, some force was used to deform the skin, and the MNE then punctured the skin and reduced the EII, which became lower than the impedance collected using the wet Ag/AgCl electrode. This result proves that the MNE is capable of puncturing the skin and collecting the bio-signal with lower contact impedance than traditional Ag/AgCl wet electrode, as well as without any pre-required skin treatment. The small wall thickness and square shape of the MNE added some flexibility to the electrode, which is useful during dynamic ECG recording because the MNE adapts to the movement and curved skin, thereby eliminating the noise due to possible drift between the skin and electrode. The electrolytic gel used in the Ag/AgCl electrode dried after some time, which introduced noise in the collected ECG signal. MNE did not use gel and resulted in a more distinguishable ECG signal, which makes it ideal for long-term bio-potential monitoring.

## References

[1] Virani S.S., Alonso A., Benjamin E.J., Bittencourt M.S., Callaway C.W., Carson A.P., Chamberlain A.M., Chang A.R., Cheng S., Delling F.N. (2020). Heart disease and stroke statistics—2020 update: A report from the American Heart Association. Circulation.

[2] Paramasivam A., Priyadharsini J.V., Raghunandhakumar S., Elumalai P. (2020). A novel COVID-19 and its effects on cardiovascular disease. Hypertens. Res..

[3] Ganatra S., Hammond S.P., Nohria A. (2020). The Novel Coronavirus Disease (COVID-19) threat for patients with cardiovascular disease and cancer. JACC CardioOncol..

[4] Searle A., Kirkup L. (2000). A direct comparison of wet, dry and insulating bioelectric recording electrodes. Physiol. Meas..

[5] O’Mahony C., Pini F., Blake A., Webster C., O’Brien J., McCarthy K.G. (2012). Microneedle-based electrodes with integrated through-silicon via for biopotential recording. Sens. Actuators A Phys..

[6] Chen K., Ren L., Chen Z., Pan C., Zhou W., Jiang L. (2016). Fabrication of micro-needle electrodes for bio-signal recording by a magnetization-induced self-assembly method. Sensors.

[7] Griss P., Tolvanen-Laakso H.K., Merilainen P., Stemme G. (2002). Characterization of micromachined spiked biopotential electrodes. IEEE Trans. Biomed. Eng..

[8] Chi Y.M., Jung T.-P., Cauwenberghs G. (2010). Dry-contact and noncontact biopotential electrodes: Methodological review. IEEE Rev. Biomed. Eng..

[9] Baek J.-Y., An J.-H., Choi J.-M., Park K.-S., Lee S.-H. (2008). Flexible polymeric dry electrodes for the long-term monitoring of ECG. Sens. Actuators A Phys..

[10] Hoffmann K.-P., Ruff R. Flexible dry surface-electrodes for ECG long-term monitoring. Proceedings of the 2007 29th Annual International Conference of the IEEE Engineering in Medicine and Biology Society.

[11] Zhou W., Song R., Pan X., Peng Y., Qi X., Peng J., Hui K.S., Hui K.N. (2013). Fabrication and impedance measurement of novel metal dry bioelectrode. Sens. Actuators A Phys..

[12] Griss P., Enoksson P., Tolvanen-Laakso H.K., Merilainen P., Ollmar S., Stemme G. (2001). Micromachined electrodes for biopotential measurements. J. Microelectromech. Syst..

[13] Gill H.S., Denson D.D., Burris B.A., Prausnitz M.R. (2008). Effect of microneedle design on pain in human volunteers. Clin. J. Pain.

[14] Luzuriaga M.A., Berry D.R., Reagan J.C., Smaldone R.A., Gassensmith J.J. (2018). Biodegradable 3D printed polymer microneedles for transdermal drug delivery. Lab Chip.

[15] Mishra R., Maiti T.K., Bhattacharyya T.K. (2018). Design and scalable fabrication of hollow SU-8 microneedles for transdermal drug delivery. IEEE Sens. J..

[16] Ren L., Jiang Q., Chen K., Chen Z., Pan C., Jiang L. (2016). Fabrication of a micro-needle array electrode by thermal drawing for bio-signals monitoring. Sensors.

[17] Kim M., Kim T., Kim D., Chung W. (2015). Curved microneedle array-based sEMG electrode for robust long-term measurements and high selectivity. Sensors.

[18] Ren L., Jiang Q., Chen Z., Chen K., Xu S., Gao J., Jiang L. (2017). Flexible microneedle array electrode using magnetorheological drawing lithography for bio-signal monitoring. Sens. Actuators A Phys..

[19] Huigen E., Peper A., Grimbergen C.A. (2002). Investigation into the origin of the noise of surface electrodes. Med. Biol. Eng. Comput..

[20] Donnelly R.F., Singh T.R.R., Tunney M.M., Morrow D.I.J., McCarron P.A., O’Mahony C., Woolfson A.D. (2009). Microneedle arrays allow lower microbial penetration than hypodermic needles in vitro. Pharm. Res..

[21] Enfield J., O’Connell M.-L., Lawlor K., Jonathan E., O’Mahony C., Leahy M. (2010). In-vivo dynamic characterization of microneedle skin penetration using optical coherence tomography. J. Biomed. Opt..

[22] Li M., Yin T., Wang Y., Du F., Zou X., Gregersen H., Wang G. (2014). Study of biocompatibility of medical grade high nitrogen nickel-free austenitic stainless steel in vitro. Mater. Sci. Eng. C.

[23] Hayashi K., Matsuguchi N., Uenoyama K., Sugioka Y. (1992). Re-evaluation of the biocompatibility of bioinert ceramics in vivo. Biomaterials.

[24] Ren L., Liu B., Zhou W., Jiang L. (2020). A mini review of microneedle array electrode for bio-signal recording: A review. IEEE Sens. J..

[25] Sun Y., Ren L., Jiang L., Tang Y., Liu B. (2018). Fabrication of composite microneedle array electrode for temperature and bio-signal monitoring. Sensors.

[26] Lim S.H., Ng J.Y., Kang L. (2017). Three-dimensional printing of a microneedle array on personalized curved surfaces for dual-pronged treatment of trigger finger. Biofabrication.

[27] Nimi N., Paul W., Sharma C.P. (2011). Blood protein adsorption and compatibility studies of gold nanoparticles. Gold Bull..

[28] Pradeep Narayanan S., Raghavan S. (2019). Fabrication and characterization of gold-coated solid silicon microneedles with improved biocompatibility. Int. J. Adv. Manuf. Technol..

[29] Dias N.S., Carmo J.P., da Silva A.F., Mendes P.M., Correia J.H. (2010). New dry electrodes based on iridium oxide (IrO) for non-invasive biopotential recordings and stimulation. Sens. Actuators A Phys..

[30] Aitzaz A., Kim J., Kim T., Park K., Cho S. (2019). Electrical characterization of pork tissue measured by a monopolar injection needle and discrete fourier transform based impedance measurement. Appl. Sci..

[31] Forvi E., Bedoni M., Carabalona R., Soncini M., Mazzoleni P., Rizzo F., O’Mahony C., Morasso C., Cassarà D.G., Gramatica F. (2012). Preliminary technological assessment of microneedles-based dry electrodes for biopotential monitoring in clinical examinations. Sens. Actuators A Phys..

[32] Johnson A.R., Caudill C.L., Tumbleston J.R., Bloomquist C.J., Moga K.A., Ermoshkin A., Shirvanyants D., Mecham S.J., Luft J.C., DeSimone J.M. (2016). Single-step fabrication of computationally designed microneedles by continuous liquid interface production. PLoS ONE.

[33] Balmert S.C., Carey C.D., Falo G.D., Sethi S.K., Erdos G., Korkmaz E., Falo L.D. (2020). Dissolving undercut microneedle arrays for multicomponent cutaneous vaccination. J. Control. Release.

